# Deterministic approach to design passive anomalous-diffraction metasurfaces with nearly 100% efficiency

**DOI:** 10.1515/nanoph-2022-0755

**Published:** 2023-03-01

**Authors:** Zhening Fang, Haipeng Li, Yan Chen, Shulin Sun, Shiyi Xiao, Qiong He, Lei Zhou

**Affiliations:** State Key Laboratory of Surface Physics, Key Laboratory of Micro and Nano Photonic Structures (Ministry of Education) and Physics Department, Fudan University, Shanghai, 200433, China; College of Information and Communication, National University of Defense Technology, Wu Han, 430000, China; Key Laboratory of Specialty Fiber Optics and Optical Access Networks, Joint International Research Laboratory of Specialty Fiber Optics and Advanced Communication, Shanghai Institute for Advanced Communication and Data Science, Shanghai University, Shanghai, 200444, China; Shanghai Engineering Research Center of Ultra-Precision Optical Manufacturing, Department of Optical Science and Engineering, School of Information Science and Technology, Fudan University, Shanghai, 200433, China

**Keywords:** anomalous reflector, deterministic approach, high efficiency, metasurfaces, surface-impedance

## Abstract

Designing perfect anomalous reflectors is crucial for achieving many metasurface-based applications, but available design approaches for the cases of extremely large bending angles either require unrealistic gain–loss materials or rely on brute-force optimizations lacking physical guidance. Here, we propose a deterministic approach to design *passive* metasurfaces that can reflect impinging light to *arbitrary* nonspecular directions with almost 100% efficiencies. With both incident and out-going far-field waves given, we can retrieve the surface-impedance profile of the target metadevice by matching boundary conditions with *all* allowed near-field modes added self-consistently and then construct the metadevices deterministically based on passive meta-atoms exhibiting local responses. We design/fabricate two proof-of-concept microwave metadevices and experimentally demonstrate that the first one achieves anomalous reflection to a 70° angle with efficiency ∼98%, and the second one can generate multiple reflected beams with desired bending angles and power allocations. Our findings pave the way for realizing high-efficiency wave-control metadevices with desired functionalities.

## 1 Introduction

Controlling electromagnetic (EM) waves at will is important for both fundamental research and practical applications. However, EM devices made by conventional materials are bulky in sizes and of low working efficiencies. Metasurfaces, planar metamaterials constructed by subwavelength microstructures (e.g., meta-atoms) with tailored EM properties arranged in certain global sequences, have recently attracted considerable attention owing to their extraordinary capabilities to control EM waves [1–4]. Many fascinating wave-manipulation effects were discovered based on metasurfaces, such as beam deflection [5–17], polarization manipulation [18–21], photonic spin-Hall effect [22–26], holograph [27–34], and meta-lens [35–37], etc. These devices are flat, ultra-thin, and highly efficient, being ideal candidates for future integration-optics applications.

**Figure 1:**
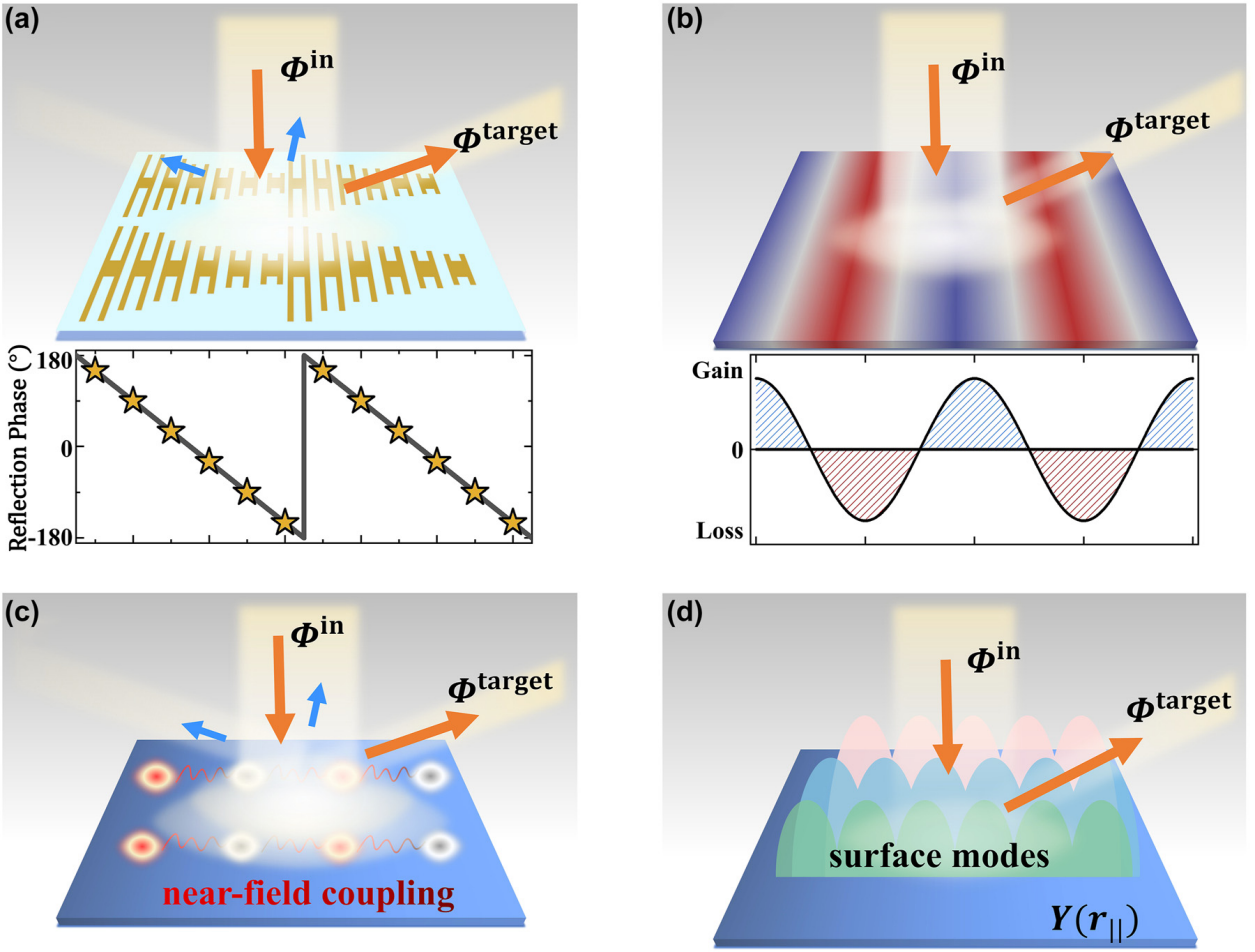
Design strategies for anomalous deflectors based on different schemes. (a) conventional gradient metasurfaces with linear phase profiles, (b) metasurfaces constructed by gain and lossy materials, (c) “*nonlocal*” metasurfaces, and (d) our passive and local metasurfaces with admittance profiles *Y*(***r***_‖_) determined with all allowed surface modes considered self-consistently. Here, blue arrows represent undesired parasitic scatterings.

Anomalous deflectors that can perfectly redirect incident light to predesigned nonspecular directions are the simplest type of metasurfaces and are the basis for realizing metadevices with more sophisticated functionalities. Unfortunately, designing *perfect* anomalous deflectors is still challenging despite of many approaches proposed. In early years, anomalous deflectors are designed to exhibit linearly varying phase profiles based upon Huygens’ principle. However, such a design principle becomes less valid as the deflection angle increases, where parasitic scatterings inevitably appear (see Figure 1(a)) [38–40]. In 2016, Alu et al. [41] proposed a new strategy to design wave-bending metasurfaces with perfect efficiencies. The key idea is to retrieve the surface impedance of target device by matching boundary conditions on the metasurface with both incident and out-going waves considered (see Figure 1(b)). However, while such a surface-impedance solution is in principle rigorous, metasurfaces thus designed inevitably exhibit certain gain and lossy responses varying in space, being extremely difficult to realize in practice. Later, some modified design approaches were proposed, based upon adding limited auxiliary surface modes in matching the boundary conditions and/or exploiting the nonlocal responses of the meta-structures (Figure 1(c)) [42, 43]. However, many of them still need brute-force optimizations to assist finding the desired realistic structures of meta-atoms [44–46] or can only be applied to certain restricted cases [47], since the nonlocal response of a certain meta-atom may also affect the designs of its adjacent meta-atoms and thus a self-consistent numerical optimization is needed. A *deterministic* design approach for realizing perfect-efficiency metadevices with diversified functionalities is highly desired.

In this paper, we establish a *deterministic* approach to design *nearly perfect* anomalous-diffraction metasurfaces constructed with passive materials exhibiting *local* responses. Different from previously proposed approaches, here we purposely add *all allowed* surface modes into the process of boundary-condition matching, with amplitudes and phases of different surface modes determined *self-consistently* requiring the device being purely passive. Retrieving the surface-impedance profile of the target device, we then construct the device with meta-atoms exhibiting purely *local* responses based on the profile *without* further structural optimizations. To illustrate the powerfulness of our approach, we design and fabricate two microwave metasurfaces and experimentally demonstrate that the first one can redirect a normally incident wave to a 70° reflection angle with an efficiency 98%, and the second one can generate two reflection beams with desired bending angles (23° and −50°) and power flux allocation (60% and 40%).

## 2 Generic theoretical framework

Figure 1(d) schematically illustrates our design approach. Consider a generic metasurface placed on the *xoy* plane (with *z* = 0), which, as shined by an incident EM wave with **Φ** ^inc^ = (***E*** ^inc^, ***H*** ^inc^), can generate a reflected beam with predesigned EM field **Φ** ^tar^ = (***E*** ^tar^, ***H*** ^tar^). Then the question is: what surface admittance distribution *Y*(***r***_‖_) should the metasurface exhibit?

To determine *Y*(***r***_‖_), we note that **Φ** ^tar^ only contains far-field (FF) information. Suppose that we can find a series of surface modes [SMs, $\Phi_{m}^{SM}=\left(E_{m}^{SM},H_{m}^{SM}\right)$ ] in the space above the metasurface, which exhibit the following characteristics: (1) they are eigen solutions of Maxwell equations in this space region; (2) they do not radiate to the FF but are bounded on the surface; and (3) they exhibit certain symmetry in accordance with the metasurface and the incident light. Thus, adding these nonradiative SMs into the total field in this space does not affect our final goal of generating the desired FF radiation but can change the near-field (NF) EM environment where the metasurface is located. Therefore, we can write the total field as $\Phi^{\text{tot}}=\Phi^{\text{inc}}+\Phi^{\text{bar}}+\underset{m}{\sum }C_{m}\Phi_{m}^{SM}$ , with {*C*_*m*_} being a set of expansion coefficients. In principle, we can find a series of metasurfaces to transform **Φ** ^inc^ into **Φ** ^tar^ in the FF, which exhibit different forms of *Y*(***r***_‖_) dictated by {*C*_*m*_} corresponding to different NF environments of the metasurfaces. Here, we choose the solution with {*C*_*m*_} satisfying the requirement that the metasurface can be realized by purely *passive* materials exhibiting *local* responses.

**Figure 2:**
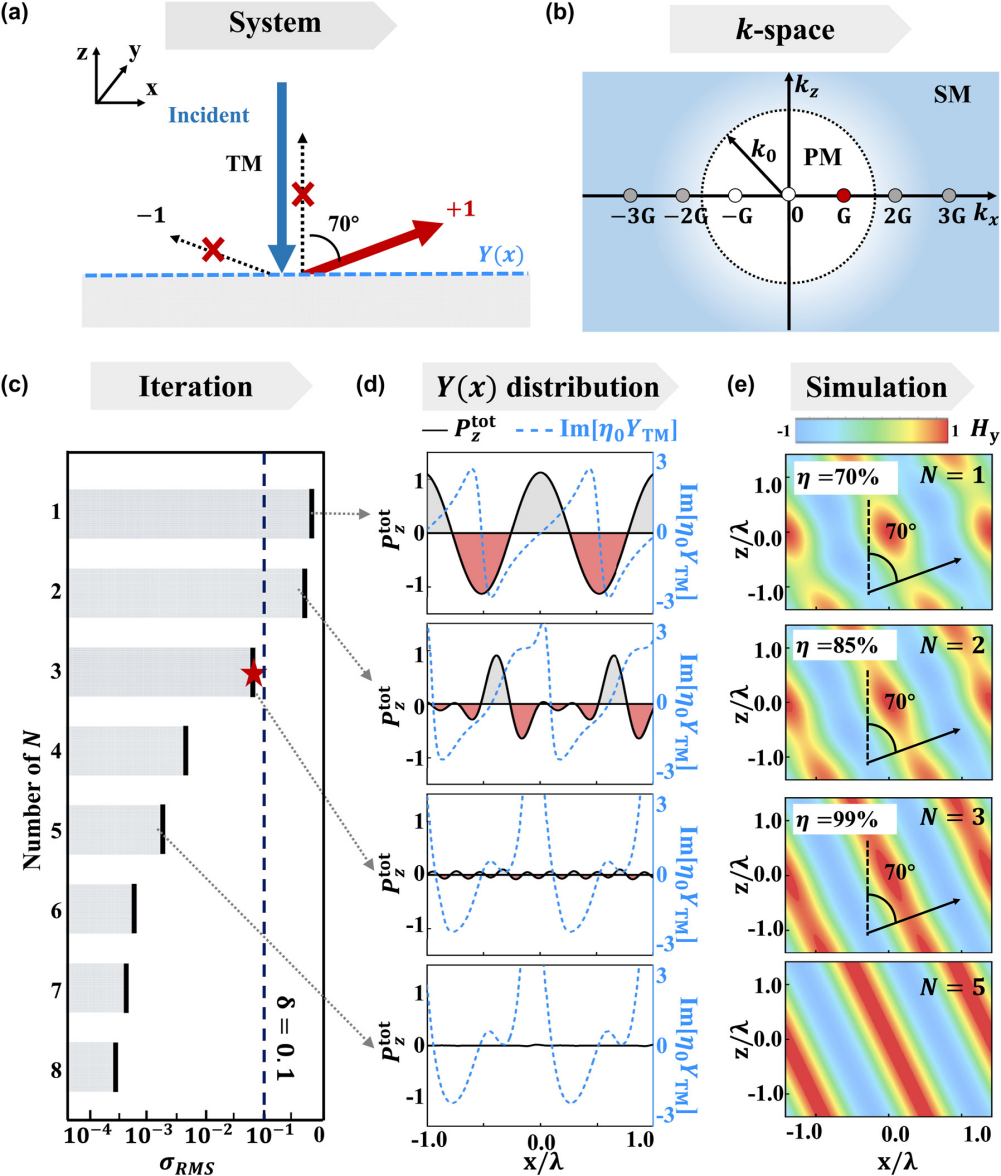
Deterministic approach of meta-deflector with arbitraray angle and perfect efficiency. (a) Schematics of a metasurface exhibiting admittance distribution *Y*_TM_(*x*) that can reflect normally incident transverse-magnetic (TM) wave to an angle of 70°, corresponding to the +1st order diffraction channel of the system. (b) Locations of different diffraction modes on the *k*_*x*_ axis, with those inside or out the circle being propagating modes (PMs) or surface modes (SMs), respectively. (c) Calculated *σ*_RMS_ with different cut-off numbers. (d) Distributions of $P_{z}^{\text{tot}}\left(x\right)\;and\;Im\left[Y_{\text{TM}}\left(x\right)\right]$ with different *N* (e.g., 1, 2, 3, and 5) considered and (e) the corresponding simulated **H**-field distributions of scattered waves on the *xoz* plane. Here, $P_{z}^{\text{tot}}\left(x\right)$ is normalized with $P_{z}^{i}$ , and *η* represents the working efficiency of the designed model metasurface.

We now illustrate the design strategy, taking the anomalous-reflection metasurface as a specific example. As shown in Figure 2(a), suppose that the incident and reflected light beams are both transverse-magnetic (TM) – polarized plane waves, propagating along *θ* ^*i*^ and *θ* ^*r*^ directions, respectively. Thus, **Φ** ^inc^ and **Φ** ^tar^ can be explicitly written as $\Phi^{\text{inc}}=\left(E^{i},H^{i}\right)=\left(-\hat{x}\eta_{0}\cos\theta^{i}\right.+\left.\hat{z}\eta_{0}\sin\theta^{i},\hat{y}\right)e^{\text{i}\left(k_{x}^{i}x-k_{z}^{i}z\right)}$ and $\Phi^{\text{tar}}=\left(E^{\text{tar}},H^{\text{tar}}\right)=A^{r}\left(\hat{x}\eta_{0}\cos\theta^{r}+\hat{z}\eta_{0}\sin\theta^{r},\hat{y}\right)e^{\text{i}\left(k_{x}^{r}x+k_{z}^{r}z\right)}$ . Here, $k_{x}^{i\left(r\right)}=k_{0}\sin\theta^{i\left(r\right)},k_{z}^{i\left(r\right)}=k_{0}\cos\theta^{i\left(r\right)}$ , *k*_0_ and *η*_0_ are the wavevector and impedance of light in vacuum, and $|A^{r}|=\frac{\sqrt{\cos\theta^{i}}}{\sqrt{\cos\theta^{r}}}$ is determined by the law of energy conservation. Now **Φ** ^*tot*^ only contains $\left\{H_{y}^{\text{tot}},E_{x}^{\text{tot}},E_{z}^{\text{tot}}\right\}$ components, we can then retrieve the surface admittance of the metasurface under design by

(1) $$Y\left(x\right)={\left.-2H_{y}^{\text{tot}}\left(x\right)/E_{x}^{\text{tot}}\left(x\right)\right|}_{z=0}.$$

It is interesting to note that *Y*(*x*) is generally a *complex* function, which consists of both real and imaginary parts. According to the Poynting theorem, we find that the real part of surface admittance Re[*Y*(*x*)] can be expressed as

(2) $$Re\left[Y\left(x\right)\right]=-4{\left.\frac{P_{z}^{\text{tot}}\left(x\right)}{|E_{x}^{\text{tot}}\left(x\right){|}^{2}}\right|}_{z=0},$$

where $P_{z}^{\text{tot}}\left(x\right)=\left(1/2\right)Re\left[E_{x}^{\text{tot}}\left(x\right)\cdot {\left(H_{y}^{\text{tot}}\left(x\right)\right)}^{*}\right]$ denotes the *z* component of Poynting vector measured on a plane right above the metasurface. Equation (2) indicates that the presence of Re[*Y*(*x*)] signifies the imbalance of energy flow at the local *x* position on the surface, which further dictates that the local constitutional material must be either lossy (with $P_{z}^{\text{tot}}<$ 0) or active (with $P_{z}^{\text{tot}}>$ 0). Hence, the criterion that our metasurface can be constructed by purely passive and lossless materials is simply

(3) $$Re\left[Y\left(x\right)\right]\equiv 0\;\;\;\;\;\;\;or\;\;\;\;\;\;\;E_{x}^{\text{tot}}\left(x\right)\equiv 0$$

at every point on the surface. In general, Equation (3) cannot be satisfied without auxiliary fields. To demonstrate this point, we take only the propagating waves into account and get

(4) $$\left\{\begin{array}{c} E_{x}^{\text{tot}}\left(x\right)=\eta_{0}\cos\theta^{r}A^{r}e^{\text{i}k_{0}\sin\theta^{r}x}-\eta_{0}\cos\theta^{i}e^{\text{i}k_{0}\sin\theta^{i}x}\;\\ H_{y}^{\text{tot}}\left(x\right)=A^{r}e^{\text{i}k_{0}\sin\theta^{r}x}+e^{\text{i}k_{0}\sin\theta^{i}x}\;\\ Y\left(x\right)=-\frac{2}{\eta_{0}}\frac{A^{r}e^{\text{i}k_{0}\sin\theta^{r}x}+e^{\text{i}k_{0}\sin\theta^{i}x}}{\cos\theta^{r}A^{r}e^{\text{i}k_{0}\sin\theta^{r}x}-\cos\theta^{i}e^{\text{i}k_{0}\sin\theta^{i}x}}\; \end{array}\right.\cdot$$

Equation (4) shows that *Y*(*x*) is a complex function as long as *θ* ^*r*^ ≠ *θ* ^*i*^, except for the special case of *θ* ^*r*^ = −*θ* ^*i*^ (i.e., the retro-reflection case) where a purely imaginary $Y\left(x\right)$ function can be found without the help of auxiliary fields (see more details in Section I of Supporting Information (SI)). Figure 1(b) plots the Re[*Y*(*x*)] distribution of a metasurface for a case of *θ* ^*i*^ = 0° and *θ* ^*r*^ = 70°, calculated with Equation (4). The periodic oscillations in Re[*Y*(*x*)] already imply that the local constitutional material constructing the metasurface must be either gain or lossy materials, which is indeed the case predicted by Ref. [41].

We now discuss how to make Equation (3) satisfied with appropriate auxiliary modes added. Assuming that our metasurface is a periodic structure with lattice constant *P*, we understand that the reflected light beam must be a linear combination of diffracted modes dictated by Bloch’s theorem. Here, we set the incident wave as the 0^th^-order mode while the reflected one is set as the 1st-order of the whole periodic system. The periodicity and two angles must satisfy the constraint 2*π*/*P* = *k*_0_ sin *θ* ^*r*^ − *k*_0_ sin *θ* ^*i*^. In this case, SMs allowed in the system are all high-order evanescent waves ( $k_{x}^{\left(m\right)}=k_{x}^{i}+m\cdot 2\pi /P$ ) with $|k_{x}^{\left(m\right)}|>k_{0}$ (see Figure 2(b)). Therefore, we get the exact field distribution of each evanescent modes as: $\Phi_{m}^{SM}=\left(E_{m}^{SM},H_{m}^{SM}\right)\;=\left(-\hat{x}\eta_{0}i\alpha_{z}^{\left(m\right)}e^{\text{i}k_{x}^{\left(m\right)}x}+\hat{z}\eta_{0}ik_{x}^{\left(m\right)}e^{\text{i}k_{x}^{\left(m\right)}x},\hat{y}C_{i}e^{\text{i}k_{x}^{\left(m\right)}x}\right)e^{\text{i}k_{x}^{\left(m\right)}x-\alpha_{z}^{\left(m\right)}z)}$ , where $\alpha_{z}^{\left(m\right)}=\sqrt{{k_{x}^{\left(m\right)}}^{2}/k_{0}^{2}-1}$ is the attenuation factor of the *m*th order of SM. Labeling these SMs according to their $\left|k_{x}^{\left(m\right)}\right|$ values, we then choose a finite number (*N*) order of SMs with amplitudes {*C*_*m*_} (with *m* = ±1, ±2, …, ±*N*) to add into the total field and obtain the total field near the metasurface, thus deriving the admittance distribution:

(5) $$\left\{\begin{array}{c} E\left(x\right)=\eta_{0}\cos\theta^{i}e^{\text{i}k_{0}\sin\theta^{i}x}-\eta_{0}\cos\theta^{r}A^{r}e^{\text{i}k_{0}\sin\theta^{r}x}-\underset{m\neq 0,\pm 1}{\sum }\eta_{0}C_{m}i\alpha_{z}^{\left(m\right)}e^{\text{i}k_{x}^{\left(m\right)}x}\;\\ H\left(y\right)=e^{\text{i}k_{0}\sin\theta^{i}x}+A^{r}e^{\text{i}k_{0}\sin\theta^{r}x}+\underset{m\neq 0,\pm 1}{\sum }C_{i}e^{\text{i}k_{x}^{\left(m\right)}x}\;\\ Y\left(x\right)=\frac{2}{\eta_{0}}\frac{e^{\text{i}k_{0}\sin\theta^{i}x}+A^{r}e^{\text{i}k_{0}\sin\theta^{r}x}+\underset{m\neq 0,\pm 1}{\sum }C_{m}e^{\text{i}k_{x}^{\left(m\right)}x}}{\cos\theta^{i}e^{\text{i}k_{0}\sin\theta^{i}x}-\cos\theta^{r}A^{r}e^{\text{i}k_{0}\sin\theta^{r}x}-\underset{m\neq 0,\pm 1}{\sum }C_{m}i\alpha_{z}^{\left(m\right)}e^{\text{i}k_{x}^{\left(m\right)}x}}\; \end{array}\right.$$

Obviously, varying the values of {*C*_*m*_} can significantly change the form of *Y*(*x*), and our task is to find a solution of {*C*_*m*_, *i* = ±1, …, ±*N*} that makes Equation (3) satisfied. Practically, Equation (3) is difficult to solve since the profile has *infinite* degrees of freedom. To make the criterion more tractable, we define:

(6) $$\sigma_{\text{RMS}}=\sqrt{\int_{-P/2}^{P/2}|P_{z}^{\text{tot}}\left(x\right){|}^{2}dx}/\sqrt{\int_{-P/2}^{P/2}|P_{z}^{\text{inc}}\left(x\right){|}^{2}dx}$$

to measure the root-mean-square (RMS) value of the imbalanced energy follow within a super cell. Therefore, Equation (3) is in principle equivalent to *σ*_RMS_ = 0. In general situations, exact solutions of {*C*_*m*_} can be found only when we add infinite number of SMs (i.e., *N* → *∞*) into the system, since Equation (3) generally has infinite number of degrees of freedom in a *continuous* space. Here, we describe a practical way to find an approximate solution with well-controlled accuracy. Our solution-searching process contains the following steps: (1) starting from a finite cut-off number *N*, using standard optimization techniques to search for the values of {*C*_*m*_} that yield the minimized value of *σ*_RMS_; (2) if Min(*σ*_RMS_) < *δ* with *δ* being the target accuracy that we aim at, then stop searching and obtain the desired{*C*_*m*_}; and (3) if not, increase the cut-off *N* and repeat the above searching procedures until a solution with satisfactory accuracy is obtained.

Figure 2(c)–(e) depict our calculation process for designing a passive metasurface that can deflect the normally incident light (*θ* ^*i*^ = 0°) to a large angle (*θ* ^*r*^ = 70°) with (nearly) 100% efficiency. By increasing the cut-off number *N* from 1 to 8, we calculate the optimized values of {*C*_*m*_} by minimizing *σ*_RMS_ in each case and obtain the distributions of $Y\left(x\right)$ for the metasurface under design by putting $\left\{C_{m}\right\}$ into Equation (5). Figure 2(c) illustrates that the *σ*_RMS_ value indeed decreases dramatically as *N* increases. Setting the accuracy threshold as =0.1, we find that adding *N* = 3 into the system (or 4 SMs in another word) is enough to ensure a convergent design meeting our accuracy requirement. Figure 2(d) depicts the distributions of $P_{z}^{\text{tot}}\left(x\right)$ and Im[*Y*(*x*)] at four representative cases with *N* = 1, 2, 3, 5, respectively. Without auxiliary SMs added (i.e., *N* = 1), we find $P_{z}^{\text{tot}}\left(x\right)$ fluctuates over *x* and its absolute values are large in certain regions, indicating that the metasurface must be constituted by lossy materials (in the region where $P_{z}^{\text{tot}}\left(x\right)<0$ ) or gain materials (in the regions where $P_{z}^{\text{tot}}\left(x\right)>0$ ). However, with more auxiliary SMs (with amplitudes $\left\{C_{m}\right\}$ appropriately adjusted) added, we find that fluctuations in $P_{z}^{\text{tot}}\left(x\right)$ are significantly suppressed. In particular, in the cases of *N* > 3, we find that $P_{z}^{\text{tot}}\left(x\right)\approx 0$ nearly everywhere, indicating that the metasurface can be constructed by purely passive materials with local responses determined by the corresponding *Y*(*x*) functions. The fact that *Y*(*x*) is (nearly) purely imaginary is also consistent with our notion that the metasurface can be constructed by local and passive materials.

To validate our design approach, we perform full-wave simulations to study the scattering of normally incident light by metasurfaces exhibiting *Y*(*x*) = Im[*Y*_*N*_(*x*)] with different values of *N*. Figure 2(e) plots the **H**-field distributions of scattered waves on the *xoz* plane in different cases. Obviously, the working efficiency of the anomalous reflector increases as a function of *N* and approaches 100% as *N* ≥ 3. In addition, the wavefront of the reflected beam becomes more flattened as *N* increases, in consistency with the enhanced anomalous-reflection efficiency. Since the complexity of the corresponding realistic structure is decided by the number of SMs included in theoretical calculation according to the Shannon sampling theorem [48–50], we decide to choose *Y*(*x*) = Im[*Y*_3_(*x*)] for final practical realization, after balancing the requirements on efficiency and simplicity.

It is interesting to compare our metasurface design scheme with the one based on conventional Huygens’ principle scheme [6, 7], in which the metasurface under design should exhibit the following surface admittance:

(7) $$Y\left(x\right)=\frac{2}{\eta_{0}\cos\theta^{i}}\frac{e^{\text{i}k_{0}\sin\theta^{r}x}+e^{\text{i}k_{0}\sin\theta^{i}x}}{e^{\text{i}k_{0}\sin\theta^{r}x}-e^{\text{i}k_{0}\sin\theta^{i}x}}$$

at each position *x* (see detailed derivation in Section II of SI). Comparing Equations (4) and (5) with Equation (7), we find that the admittance profile retrieved in Ref. [41] (Equation (4)) has correctly considered the local-field correction due to the desired radiated FF, and our newly developed formula (Equation (5)) further considers the local-field corrections due to NFs contributed by *all possible* SMs allowed by the system. With more possible modes added, it is natural to expect that our scheme can yield better results than previous trials.

**Figure 3:**
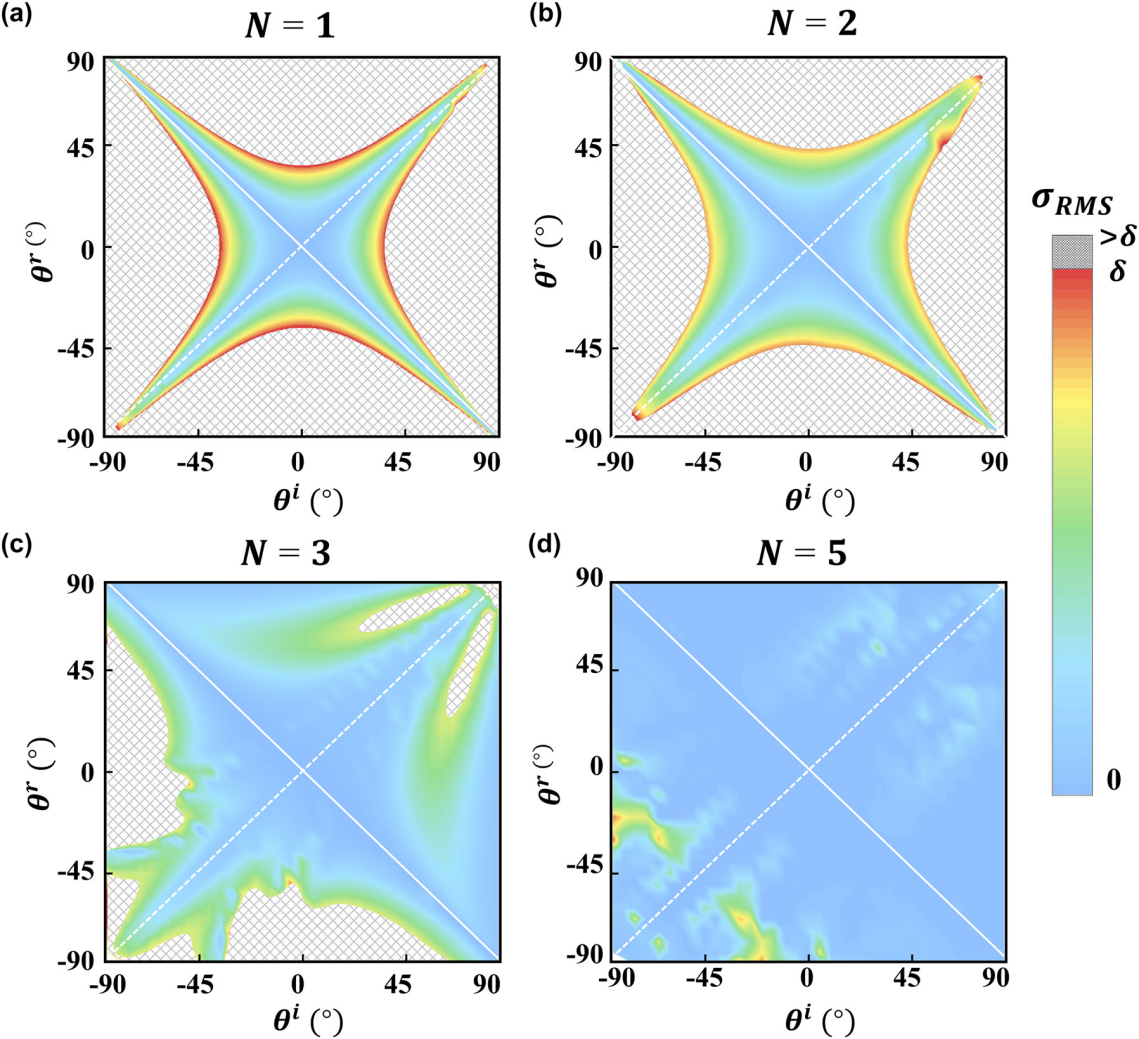
*σ*_RMS_ of the designed anomalous-reflection metasurface versus *θ* ^*i*^ and *θ* ^*r*^. The figures are calculated with (a) *N* = 1, (b) *N* = 2, (c) *N* = 3, and (d) *N* = 5 considered, correspondingly. Here, we set *δ* = 0.1. Dashed line and solid line denote the specular reflection mode and the retro-reflection mode, respectively, both of which are the rigorous solutions to Equation (3) without the help of auxiliary fields.

We now discuss the performance of our design scheme for different {*θ* ^*i*^, *θ* ^*r*^} combinations. Figure 3 depicts how the theoretically calculated *σ*_RMS_ varies against *θ* ^*i*^ and *θ* ^*r*^ with different number of SMs added. In each case, we always find that *σ*_RMS_ = 0 in the case of specular reflection (*θ* ^*i*^ = *θ* ^*r*^, dashed line), or retro-reflection (*θ* ^*i*^ = −*θ* ^*r*^, solid line), as expected. Meanwhile, the convergence area (i.e., with *σ*_RMS_ < *δ*) in the *θ* ^*i*^ – *θ* ^*r*^ phase diagrams continuously expands as *N* increases, indicating that adding more SMs can indeed help speed up obtaining convergent solutions for highly mismatched incidence/deflection angles. Practically, we find that setting *N* = 5 in our scheme is enough to ensure finding appropriate admittance profiles for metasurfaces with arbitrary anomalous-reflection abilities.

## 3 Meta-atom designs

We now design a set of meta-atoms that are suitable to construct our metasurfaces with admittance distributions given in Equation (5). Since our scheme has ensured that the final $Re\left[Y\left(x\right)\right]$ function is negligible, we only consider the $Im\left[Y\left(x\right)\right]$ function in matching the boundary conditions. In practical designs, it is more convenient to sort out meta-atoms from the required reflection properties (including reflection phase and amplitude). Connecting the admittance Im(*Y*) with reflection coefficient *r* of a given surface and assuming that the meta-atoms exhibit local responses, we find that the meta-atom located at the position *x* should exhibit the following reflection coefficient

(8) $$r\left(x\right)=\frac{2-i\eta_{0}Im\left[Y\left(x\right)\right]}{2+i\eta_{0}Im\left[Y\left(x\right)\right]},$$

determined by the admittance value required at this very position *x*. Equation (8) can be further re-written as *r*(*x*) = e^i*φ*(*x*)^ with reflection amplitude being 100% and reflection phase given by *φ*(*x*) = −2 arctan[*iη*_0_ *Y*(*x*)]. Therefore, our task is to find a series of meta-atoms that can perfectly reflect EM waves with different reflection phases and exhibit local responses.

**Figure 4:**
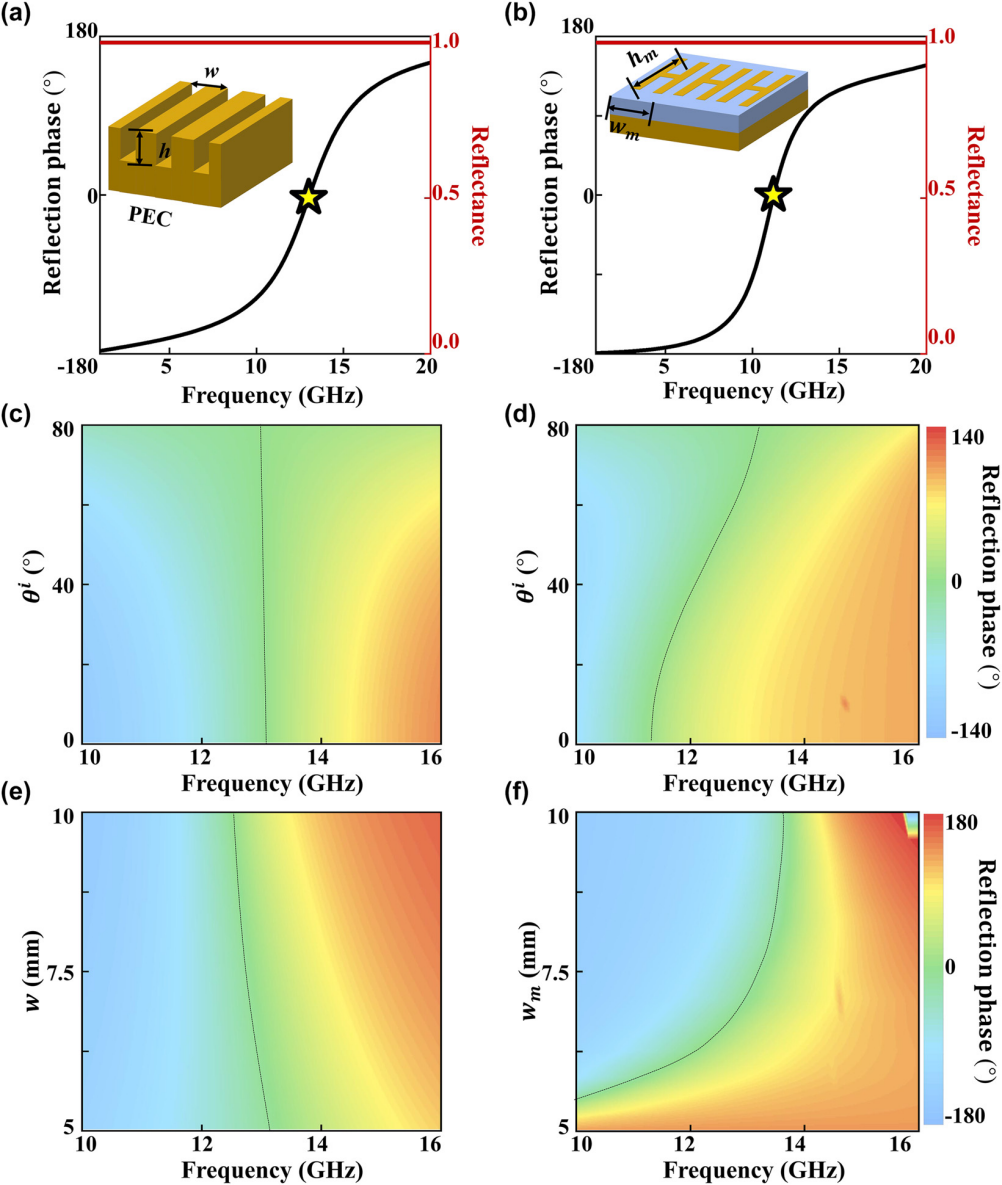
FEM-computed normal-incidence spectra of reflection amplitude and phase of a periodic metasurface consisting of (a) groove meta-atoms (see inset) or (b) MIM meta-atoms (see inset) with the following geometrical parameters: *h* = 5.23 mm, *w* = 6 mm, *h*_*m*_ = 3.05 mm, and *w*_*m*_ = 6 mm, respectively. FEM-computed reflection phase versus frequency and incident angle of a metasurface consisting of periodic arrays of (c) groove meta-atoms or (d) MIM meta-atoms. FEM-computed reflection phase versus frequency and periodicity of a metasurface consisting of periodic arrays of (e) groove meta-atoms or (f) MIM meta-atoms. Black dashed lines in (c–f) denote the zero-reflection-phase positions corresponding to the resonant frequencies of the systems.

We find that the **groove** structure, where a groove is etched on a metallic plate (see inset to Figure 4(a)), is the suitable meta-atom satisfying all above requirements. The continuous metallic film on the back can ensure total reflection of incident EM wave, while changing the height *h* of the groove can modify the resonant mode supported by the structure, which in turn, changes the reflection phase drastically [51]. More importantly, compared with the metal–insulator–metal (MIM) meta-atoms (see Figure 4(b)) that can also perfectly reflect EM waves with tailored reflection phases [52], the **groove** meta-atoms exhibit more localized responses to external illuminations, manifested by much reduced mutual couplings between neighboring meta-atoms. To illustrate this property, we numerically examine how the resonant modes supported by the **groove** or MIM meta-atoms, both being repeated in *xy*-planes forming periodic metasurfaces, evolve as a function of the incident angle *θ*_*i*_ (Figure 4(c) and (e)) or the width of the meta-atoms *w* (*w*_*m*_) for the periodic structure (Figure 4(d) and (f)), respectively. As either *θ*_*i*_ or *w* (*w*_*m*_) changes, while the resonant mode is hardly changed in the **groove structure** case (Figure 4(c) and (e)), the same thing is not true for the MIM case (Figure 4(d) and (f)). Noting that angular dispersion of a metasurface is ultimately determined by the coupling between neighboring meta-atoms [14, 53], we reach the conclusion that mutual-coupling issue is weaker in the **groove structure** case than in the MIM case. These results confirm that the **groove** meta-atoms exhibit highly localized optical responses under external excitations, which are highly desired for constructing our metasurfaces in a *deterministic* way.

Figure 4(a) depicts the numerically computed spectra of reflection amplitude and phase of a periodic array of a typical meta-atom with *w* = 6 mm and *h* = 3.05 mm. Clearly, we find that the reflection amplitude is always 100%, while the reflection phase changes from −*π* to *π* continuously as frequency increases, due to the cavity resonant mode at *f* = 13.3 GHz supported by the groove. Fixing the working frequency at 13.3 GHz, we employ FEM simulations to establish the relation between the reflection phase of the meta-atom and the parameter *h*, which facilitates our designs in the remaining part of this paper (see more details in Section III of SI).

## 4 Experimental demonstration of an anomalous deflector with large bending angle and nearly perfect efficiency

**Figure 5:**
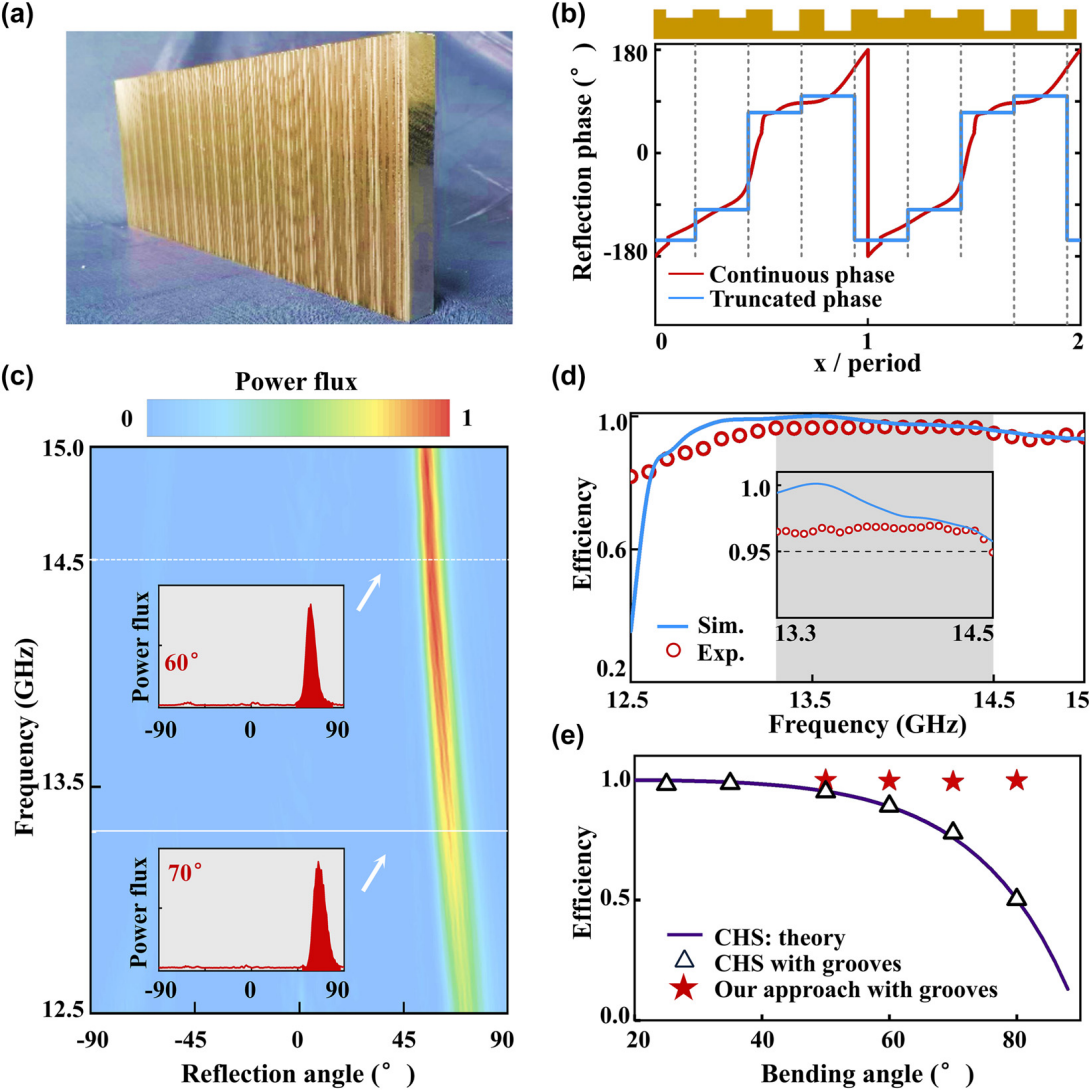
Experimental demonstration of meta-deflector with bending angle of 70°. (a) Picture of the fabricated anomalous meta-deflector composed by groove meta-atoms. (b) Reflection-phase distribution of the metadevice obtained with our theory (red line) and its truncated version (blue line) based on which the realistic structure is designed. Top panel depicts the side-view picture of the realistic sample. (c) Measured normalized scattered power flux versus reflection angle and frequency, as the metasurface is shined by normally incident waves at different frequencies. Two insets illustrate the angular distributions of measured scattered power flux at 13.3 GHz and 14.5 GHz, respectively. (d) Working efficiency of the anomalous meta-deflector as a function of frequency, obtained by experiments (red circle) and simulations (blue line). Inset depicts the zoom-in view of the efficiency spectra within the shaded frequency range. (e) Numerically simulated working efficiencies of anomalous meta-deflector aiming for different bending angles, with the metasurfaces as model systems designed by CHS (solid line), as realistic systems composed by groove meta-atoms according to the CHS design (triangles), and as realistic systems composed by groove meta-atoms according to our new scheme (stars).

We first employ the proposed scheme to design an anomalous reflector that can deflect a normally incident TM-polarized wave to the angel of 70° with nearly perfect efficiency at the frequency 13.3 GHz. Retrieving the admittance profile *Y*(*x*) of the target metadevice with 4 SMs added self-consistently (*N* = 3), we employ Equation (8) to obtain the required phase distribution *φ*(*x*) of the metasurface (red curve in Figure 5(b)). Calculations on a model system possessing the retrieved *Y*(*x*) profile show that the device exhibits a working efficiency *η* = 99.9%. Set the working frequency at 13.3 GHz, we follow the design strategy described in Section 3 to sort out 4 **groove** meta-atoms with different *h* based on the discretized reflection phases (see blue curves in Figure 5(b)) and use them to form our metadevice. Figure 5(a) shows the photograph of a fabricated sample containing 20 meta-atoms in total, with a side-view picture shown in the upper panel of Figure 5(b). It is interesting to note that the desired *φ*(*x*) profile is quite different from the gradient phase profile according to Huygens’ principle, due to the local-field corrections discussed in Section II of SI.

We then experimentally characterize the performance of the fabricated metadevice. In our measurements, we adopt a horn antenna as a source to normally illuminate the fabricated sample and put another one on a circular track with a radius of 1 m to measure the angular distribution of scattered power flux. Both antennas are connected to a vector network analyzer. Figure 5(c) plots the measured scattered power flux as a function of frequency and reflection angle. Here, the reference is defined as the power flux reflected by a flat metallic mirror (with the same size as our metadevice) under the same excitation condition. Clearly, the anomalous-reflection mode takes nearly all scattered power, while parasitic scatterings are significantly suppressed within the whole frequency band (12.5–15.0 GHz). Meanwhile, the measured peak deflection angle *θ* ^*r*^ is a decreasing function of frequency *f*, following the diffraction law $\theta^{r}=\arcsin\left(\frac{c_{0}}{fp}\right)$ .

We quantitatively estimate the working efficiency of the fabricated metadevice, defined as the ratio between the integrated power carried by the anomalous reflection beam and that by the impinging beam. Insets to Figure 5(c) depict the measured angular distributions of the power flux of scattered beam at two particular frequencies 13.3 GHz and 14.5 GHz, from which we can quantitatively evaluate the working efficiencies based on the power integrations. We depict the evaluated working efficiencies of our metadevice at different frequencies as solid circles in Figure 5(d). We also employ FEM simulations to study the scattering patterns of the metadevice, from which we retrieve the working efficiencies of the device at different frequencies, and depict them as a solid line in Figure 5(d). Experimentally retrieved working efficiencies are generally in excellent agreement with numerically retrieved ones. In particular, the experimental efficiencies of our metadevice are found to exceed 95% within 13.3–14.5 GHz, unambiguously demonstrating the broadband high-efficiency performance of the device. We note that the imperfect working efficiency obtained in our experiment is due to imperfections in our experimental characterizations, including the finite-size effect of the fabricated metasurface and the nonideal incident wave. Such issues are generally irrelevant with the design strategy to realize the metasurfaces but rather solvable via improving the experimental conditions.

To illustrate its powerfulness, we further design anomalous reflectors with bending angle changing from 50° to 80° under normal incidence and then numerically estimate their working efficiencies. It should be noted that the working frequencies of these metadevices are tied with their target bending angle *θ* ^*r*^ as long as we fix the value of super periodicity *P*. Solid stars in Figure 5(e) are the calculated efficiencies of these metadevices, which are all very close to 100%. In comparison, we also use the **groove** meta-atoms to construct metadevices exhibiting different deflection angles, designed with the conventional Huygens’ principle scheme (see Section IV of SI for details). Numerical evaluations on these realistic structures show that their working efficiencies (black hollow triangles) follow nicely with theoretical prediction (purple solid line) based on Equation (8), deviating quickly from 100% as the target deflection angle increases. This reinforces our notation that metadevices designed with the conventional Huygens’ principle scheme inevitably exhibit low working efficiencies for large bending angles, since such a design approach neglects local field corrections. We emphasize that our design scheme can also be adopted to realize meta-deflectors for arbitrary bending angles (e.g., even near the grazing angle) with almost perfect efficiency.

**Figure 6:**
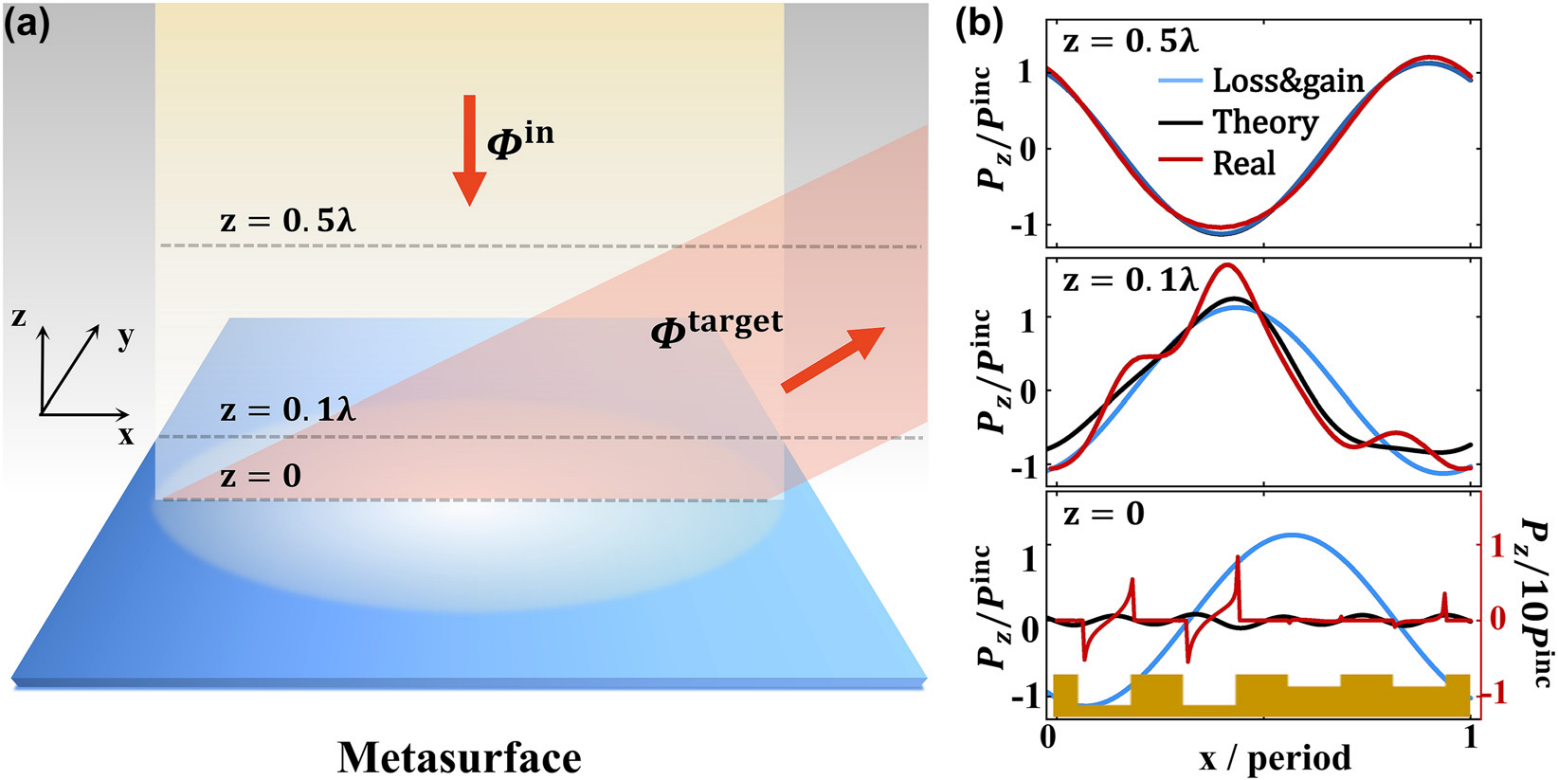
Power flux analysis for the meta-deflector with bending angle of 70°. (a) Schematics of the investigated system. (b) Normalized distributions of $P_{z}^{\text{tot}}\left(x\right)$ on 3 horizontal planes at *z* = 0, 0.1*λ*, and 0.5*λ* above the metasurface shined by normally incident wave. Blue lines represent the case that the metasurface is the loss & gain metasurface, black lines represent the case that the metasurface is a model system designed with our approach, and red lines represent the case that the metasurface is a realistic system composed by groove meta-atoms [see inset of the lower panel in (b)]. Red line in the bottom panel represents the result multiplied by a factor of 1/10.

To reveal the underlying physics, we compare the power flux distributions in the space above the metasurfaces designed with different approaches, all under illuminations of the same normally incident EM waves (see Figure 6(a)). We consider three different systems, which are the model metasurfaces with admittance profiles given by the gain & loss scheme and our newly proposed theory, and the realistic metasurface composed by **groove** meta-atoms. All these metasurfaces are designed for 70° bending angle under normal incidence. Figure 6(b)–(d) compare the distributions of $P_{z}^{\text{tot}}\left(x\right)$ on three planes with different *z* above the metasurfaces. We find that the $P_{z}^{\text{tot}}\left(x\right)$ distribution only exhibits a lateral shift as *z* varies in the case of gain & loss metasurface (blue lines in Figure 6(b)), caused by the interference between incident wave and anomalously reflected wave, which possesses a phase factor $e^{\text{i}k_{x}x}$ . In contrast, $P_{z}^{\text{tot}}\left(x\right)$ changes significantly as *z* varies in the case of our metasurface (black lines in Figure 6(b)). Such an obvious difference is induced by the additional SMs added in our scheme, which significantly changes the total wave pattern and thus the final $P_{z}^{\text{tot}}\left(x\right)$ distribution. As *z* decreases, auxiliary modes considered in our scheme exhibit stronger fields due to their evanescent natures and thus the modification on $P_{z}^{\text{tot}}\left(x\right)$ becomes more dramatic. In particular, on the *z* = 0 plane right above the metasurface, $P_{z}^{\text{tot}}\left(x\right)$ is nearly 0 everywhere in the case of our metasurface, consistent with the proposed design criterion Equation (3). The evolution of $P_{z}^{\text{tot}}\left(x\right)$ over *z* well reveals the crucial role played by the additional SMs added in our design scheme, which help match the boundary conditions on the metasurface yet keep the far fields as desired. Finally, we also compare $P_{z}^{\text{tot}}\left(x\right)$ in the cases of model metasurface and its corresponding realistic structure. These two $P_{z}^{\text{tot}}\left(x\right)$ distributions essentially exhibit the same behaviors except for certain fluctuations in the realistic case, due to strong near fields induced around the corners of realistic meta-atoms. Nevertheless, we find that the average of $P_{z}^{\text{tot}}\left(x\right)$ over a single meta-atom in the realistic-sample case (red line) is close to the value of $P_{z}^{\text{tot}}\left(x\right)$ in the model case (black line), both being nearly zero, in consistency with the effective medium theory.

## 5 Application: metasurface enabling multiple beam generation with predesigned property

**Figure 7:**
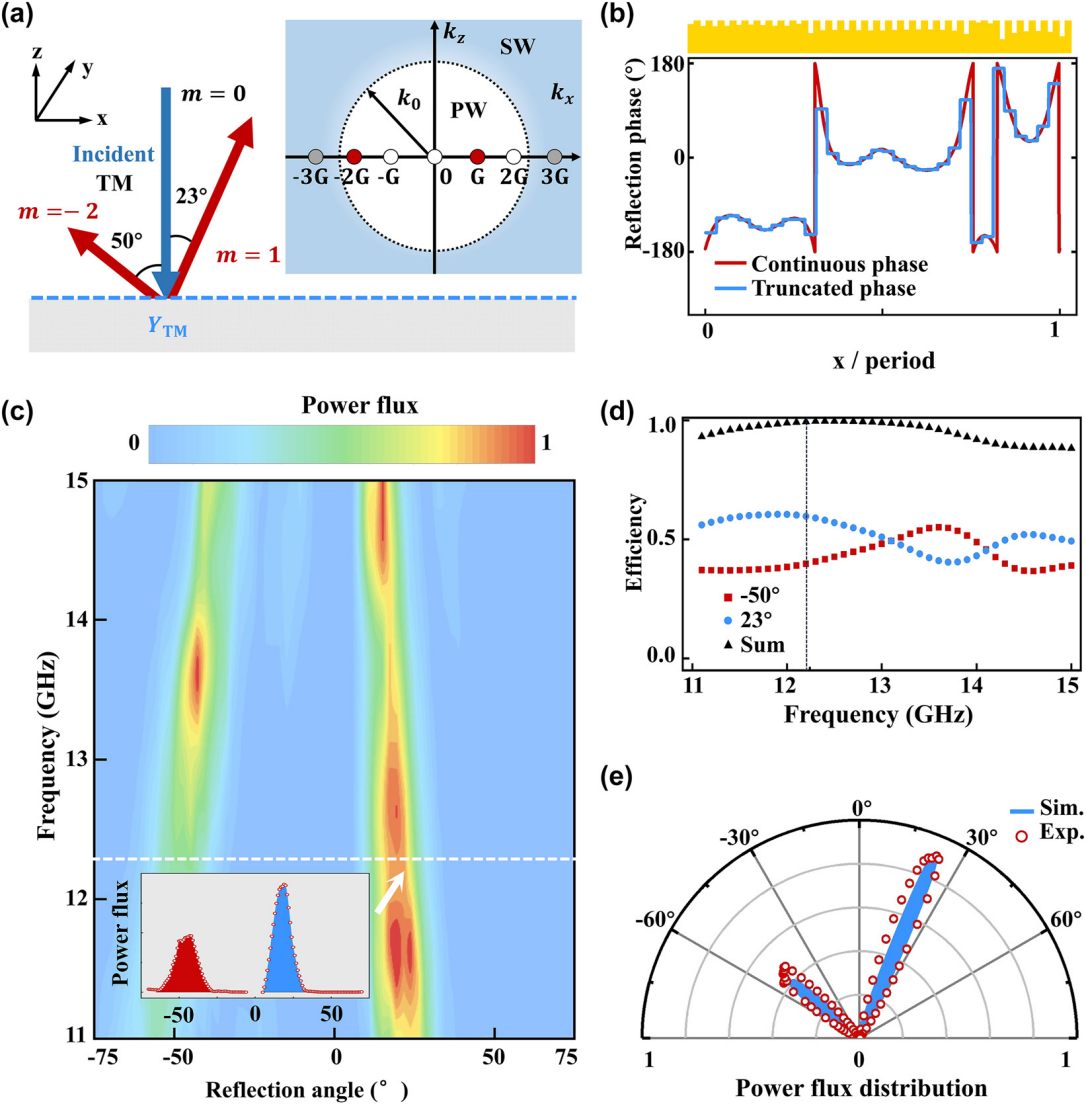
Experimental demonstration of metasurface-based multiple beam generator. (a) Left: Schematics of the multiple-beam generator designed with our strategy. Right: Properties of diffraction modes with different orders. (b) Red line represents the reflection phase profile of the metadevice exhibiting the reflection functionality as shown in (a), designed by our scheme. Blue line represents a truncated version of red line, based on which the realistic metadevice is achieved. Top panel depicts the side-view picture of the realistic sample. (c) Measured normalized scattered power flux versus detection angle and frequency, as the metasurface is shined by normally incident waves at different frequencies. Inset illustrates the measured angular distribution of scattered power flux at 12.2 GHz. (d) Blue circles and red squares represent the calculated power efficiencies of two reflected beams generated by our metadevice, as shined by normally incident waves at different frequencies. Black triangles represent the sum of power efficiencies carried by two modes. (e) Measured (red hollow circle) and calculated (blue line) normalized far-field scattering patterns of our metadevice, as shined by normally incident wave at 12.2 GHz.

Our scheme is so general that it can also be extended to design other metadevices for realizing more complex wavefront controls with nearly 100% efficiencies. As an illustration, we design a metasurface that can reflect normally incident EM wave to two different directions (*θ* ^*r*1^ = 23° and *θ* ^*r*2^ = −50°) with efficiencies 60% and 40%, respectively, as schematically shown in Figure 7(a). We purposely set the super periodicity as $P=\lambda_{0}/\left|\sin\theta^{r1}-\sin\theta^{i}\right|$ so that the deflection angles for +1 and −2 diffraction modes are just *θ* ^*r*1^ = 23° and *θ* ^*r*2^ = −50°, respectively, according to Bloch’s theorem. With the target scattering pattern known, we can thus write out the total EM fields in the region above the metasurface. Specifically, the parallel components of **E** and **H** fields are:

(9) $$\left\{\begin{array}{c} H_{\|}=e^{\text{i}k_{0}\sin\theta^{i}x}+A^{r1}e^{\text{i}k_{0}\sin\theta^{r1}x}+A^{r2}e^{\text{i}k_{0}\sin\theta^{r2}x}+\underset{m\neq 0,\pm 1,\pm 2}{\sum }C_{i}e^{\text{i}k_{x}^{\left(m\right)}x}\;\\ E_{\|}=\eta_{0}\left[\cos\theta^{i}e^{-ik_{0}\sin\theta^{i}x}-\cos\theta^{r1}A^{r1}e^{-ik_{0}\sin\theta^{r1}x}-\cos\theta^{r2}A^{r2}e^{-ik_{0}\sin\theta^{r2}x}\right.\;\\ \left.-\underset{m\neq 0,\pm 1,\pm 2}{\sum }C_{i}i\alpha_{z}^{\left(m\right)}e^{-ik_{x}^{\left(m\right)}x}\right]\; \end{array}\right.$$

where the expansion coefficients of two desired scattering FF modes are $|A^{r1}|=\frac{\sqrt{3}}{\sqrt{5\cos\left(\theta^{r1}\right)}}$ and $|A^{r2}|=\frac{\sqrt{2}}{\sqrt{5\cos\left(\theta^{r2}\right)}}$ , respectively, dictated by the pre-conditions and the law of energy conservation, and {*C*_*m*_} are a set of coefficients of the NF SMs added in our scheme.

We then follow the design strategy described in Section 2 to determine the expansion coefficients {*C*_*m*_} to complete our design. Setting *N* = 8, we obtain a metasurface design exhibiting satisfactory accuracy, with reflection phase profile shown as a red line in Figure 7(b). We then truncate the profile phase into 32 sub cells (blue line in Figure 7(b)) and sort out the corresponding **groove** meta-atoms based on the desired phases of each subcells. We next fabricate out the sample according to the design (see Section III of SI for the detailed parameters), and a side-view picture is shown in the upper inset to Figure 6(b). With the fabricated metasurface at hand, we then experimentally measure its reflection patterns at different frequencies under normal incidence, following the characterization scheme described in last section. Figure 7(c) depicts the measured normalized power flux as the function of reflection angle and frequency, with the inset showing the angle distribution measured at the frequency 12.2 GHz corresponding to the dashed line. From the measured data, we can easily retrieve the power flux taken by two scattered modes and calculate their efficiencies with the reference signal taken as that reflected by a flat metallic surface. We find that at the working frequency 12.2 GHz, these two scattered modes take the power efficiencies 59.7% and 39.7%, respectively, in nice agreement with the predesigned conditions. The sum of these efficiencies is generally close to 100% and reaches 99.4% at 12.2 GHz in particular, indicating that the parasitic scatterings are negligible. We also note that the working efficiencies oscillate with the frequency of input wave, which is due to our frequency-dependent design strategy and the intrinsic dispersion properties of our constitutional meta-atoms. Figure 7(e) compares the normalized scattering patterns of our metadevice under normal-incidence illumination at 12.2 GHz, obtained by experiments and FEM simulations. Nice agreement is noted between these two patterns.

## 6 Conclusions

To summarize, we established a deterministic approach to design purely passive metasurfaces that can reflect incident light to arbitrary nonspecular directions with nearly perfect efficiencies. With both incident and out-going far-field waves known, we purposely add all allowed surface waves into the process of boundary-condition matching and determine their coefficients under the requirement that the device remains purely passive and local. Retrieving the surface-impedance distribution of the target device, we employ **groove** meta-atoms to construct realistic metasurfaces according to the impedance profiles. Two metadevices are designed/fabricated and experimentally characterized, one enabling perfect anomalous reflection to a large bending angle and another splitting normally incident beam to two anomalous reflection channels with predesigned efficiencies. Our results open the door to realize high-efficiency wave-control metadevices with diversified functionalities.

## Supplementary Material

Supplementary Material Details
