# Supplementary material for: Deterministic approach to design passive anomalous-diffraction metasurfaces with nearly 100% efficiency
Source: Nanophotonics. 2023 Mar 1;12(13):2383–96. doi: 10.1515/nanoph-2022-0755 (PMC11501116; doi:10.1515/nanoph-2022-0755)
Supplement: Supplementary file 1 — Supplementary Material Details [file j_nanoph-2022-0755_suppl_001.docx]

***Supplementary information for***

**Deterministic approach to design passive** **anomalous-diffraction metasurfaces with almost 100% efficiency**

**Zhening Fang^1^, Haipeng Li^2^, Yan Chen^3^, Shulin Sun^4^, Shiyi Xiao^3,*^,**

**Qiong He^1,*^，and Lei Zhou^1,*^**

^1^ State Key Laboratory of Surface Physics, Key Laboratory of Micro and Nano Photonic Structures (Ministry of Education) and Physics Department, Fudan University,
Shanghai, China, 200433

^2^ College of Information and Communication, National University of Defense Technology, Wu Han 430000, China

^3^ Shanghai University, Key Laboratory of Specialty Fiber Optics and Optical Access Networks, Joint International Research Laboratory of Specialty Fiber Optics and Advanced Communication, Shanghai Institute for Advanced Communication and Data Science, Shanghai, China, 200444

^4^ Shanghai Engineering Research Center of Ultra-Precision Optical Manufacturing, Department of Optical Science and Engineering, School of Information Science and Technology, Fudan University, Shanghai 200433, China

**List of contents:**

**Section I** – **Power flux analysis for retro-reflection case**

**Section II** – **Detailed** **derivation for Equation 7 in the main text**

**Section III** – **Details for the realistic structure design of our meta-devices**

**Section IV** – **Details information for Figure 5(e) in the main text**

**Section I – Power flux analysis for retro-reflection case**

Following the discussions in Section II in the main text, for the case related to Eq. (4), we can write out the total vertical power flux (without considering the near field) as:

$$P_{z}^{\text{tot}}(x)=\mathrm{Re}[\frac{\eta_{0}}{2}(A^{r}e^{-ik_{0}\sin\theta^{r}x}+e^{-ik_{0}\sin\theta^{i}x})(\cos\theta^{r}A^{r}e^{ik_{0}\sin\theta^{r}x}-\cos\theta^{i}e^{ik_{0}\sin\theta^{i}x})]$$

$$\propto(A^{r}[\cos(k_{0}\sin\theta^{r}x)-i\sin(k_{0}\sin\theta^{r}x)]+[\cos(k_{0}\sin\theta^{i}x)-i\sin(k_{0}\sin\theta^{i}x)])(\cos\theta^{r}A^{r}[\cos(k_{0}\sin\theta^{r}x)+i\sin(k_{0}\sin\theta^{r}x)]-\cos\theta^{i}[\cos(k_{0}\sin\theta^{i}x)+i\sin(k_{0}\sin\theta^{i}x)]$$

$=A^{r}(\cos\theta^{r}-\cos\theta^{i})\cos[k_{0}x(\sin\theta^{r}-\sin\theta^{i})]$ . (S1.1)

It is obvious that the solutions for $P_{z}^{\text{tot}}(x)\equiv0$ are $\theta^{r}=\theta^{i}$, or $\theta^{r}=-\theta^{i}$. The former one corresponds to the trivial specular reflection, while the latter corresponds to the retro-reflection. Putting $\theta^{r}=-\theta^{i}$ into the field distribution assumed, we find that the local field is written as:

$H_{y}^{\text{tot}}(x)=\frac{\eta_{0}}{2}(e^{ik_{0}\sin\theta^{i}x}+e^{-ik_{0}\sin\theta^{i}x})=\eta_{0}\cos(k_{0}\sin\theta^{i}x)$,

$E_{x}^{\text{tot}}(x)=\cos\theta^{i}(e^{ik_{0}\sin\theta^{i}x}-e^{-ik_{0}\sin\theta^{i}x})=2i\cos\theta^{i}\sin(k_{0}\sin\theta^{i}x)$. (S1.2)

We notice that the local electric and magnetic field are completely out of phase, contributing 0 power flux along the z-axis, which is the reason why retro-reflection is also a rigorous solution without the help of auxiliary field.

**Section II –** **Detailed derivation for Equation 7**


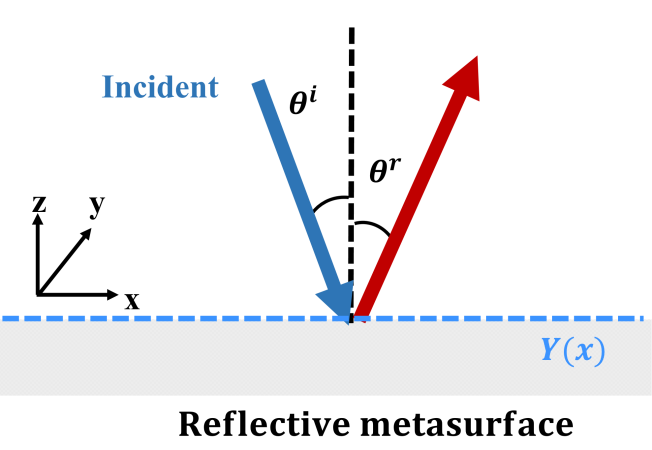


Figure S1. Schematics of a metasurface with input wave with incident angle $\theta^{i}$.

We start from calculating the total field where a metasurface is shined with TM plane wave with an incident angle $\theta^{i}$, as shown in Figure S1. To bend the incident wave into a desired reflection angle $\theta^{r}$, the metasurfaces based on the conventional Huygens-principle scheme should exhibit the linear reflection phase distribution, whose H field can be written as following:

$\left\{ \begin{aligned} \vec{H}_{y}^{i}(x)=e^{ik_{0}\sin\theta^{i}x}e^{-i\omega t}\hat{y} \\ \vec{H}_{y}^{r}(x)=e^{ik_{0}\sin\theta^{r}x}e^{-i\omega t}\hat{y}=\vec{H}_{y}^{inc}e^{i\varphi x} \end{aligned} \right.$, (S2.1)

where $\varphi=k_{0}(\sin\theta^{r}-\sin\theta^{i})$ is the linear phase distribution. By picking out a specific ultra-small meta-atom placed at $x=x_{0}$ and duplicating it infinitely along the x-axis, we construct another surface. As required in Equation (S2.1), the new surface satisfies that when shined upon incident wave with incident angle $\theta^{i}$, the reflectance must be set to:

$r =e^{i\varphi x_{0}}$. (S2.2)

Therefore, the total field for the new system is written as:

$\left\{ \begin{matrix} \vec{H}_{y}^{i}(x)=e^{ik_{0}\sin\theta^{i}x}e^{-i\omega t}\hat{y} \\ \vec{E}_{x}^{i}(x)=-\eta_{0}\cos\theta^{i}e^{ik_{0}\sin\theta^{i}x}e^{-i\omega t}\hat{x} \\ \vec{H}_{y}^{r}(x)=e^{ik_{0}\sin\theta^{r}x}e^{-i\omega t}\hat{y} \\ \vec{E}_{x}^{r}(x)=e^{i\varphi x_{0}}\eta_{0}\cos\theta^{i}e^{ik_{0}\sin\theta^{i}x}e^{-i\omega t}\hat{x} \end{matrix} \right.$,. (S2.3)

Recalling Equation (1) in the main text, we get the local surface admittance for such a surface:

$Y(x=x_{0}) =\frac{2}{\eta_{0}\cos\theta^{i}}\frac{e^{ik_{0}\sin\theta^{r}x_{0}}+e^{ik_{0}\sin\theta^{i}x_{0}}}{e^{ik_{0}\sin\theta^{r}x_{0}}-e^{ik_{0}\sin\theta^{i}x_{0}}}$. (S2.4)

By applying such process to each point along the required metasurface, we get Equation (7) in the main text.

**Section III – Details for the realistic structure design of our meta-devices**

1. **Truncation of the reflection phase into discretized step function**

In order to realize the calculated reflection phase profile with finite-sized meta-atoms, the phase profile need be truncated into step function fitting with the size of meta-atoms. We use the truncated surface admittance and impedance to design our metasurface with necessary numbers of meta-atoms based on FEM-simulation, without violating the high working efficiency property. Following Shannon sample theorem [1-3], we start from the continuous profile, truncate the curve into many atoms, and slowly decrease the number of meta-atoms in a period (Figure S4). We use the truncated surface admittance and impedance to perform simulation with FEM, confirming that the truncation does not affect the resulting efficiency. According to our approach, we only need to consider $\pm3$ order modes for the anomalous deflector case (with bending angle of 70$^{\circ}$), and designed realistic metasurface with only 4 different groove structures as show in Figure 5(b) in the main text. For the multiple-reflected beam generator with much more high-order modes considered, we design our metasurface with 32 different groove structures to fulfil the phase profile requirement, as shown in Figure 7(b) in the main text.


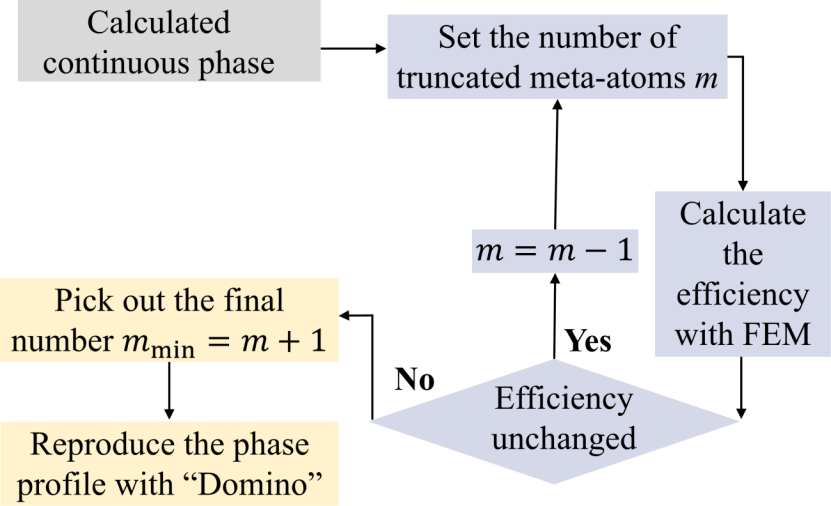


Figure S2. The flow chart to minimize the number of meta-atoms in a period for each specific design.

1. **Structure design for anomalous deflector with** $\boldsymbol{70^{\circ}}$ **bending angle**


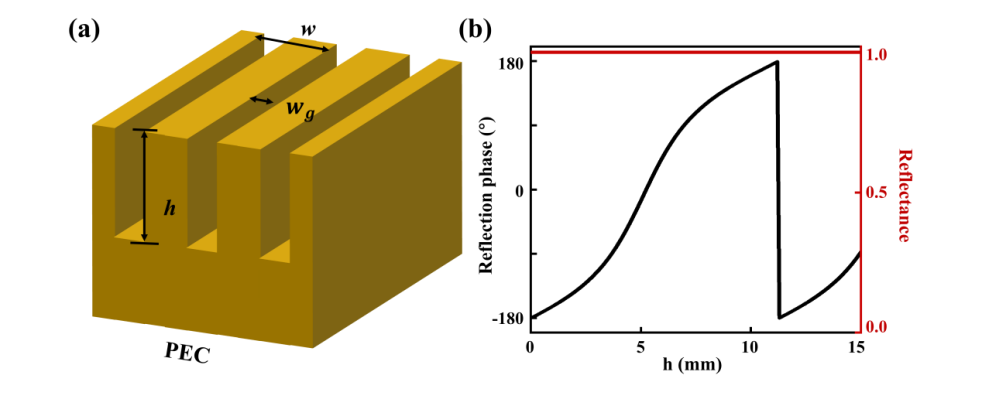


Figure S3. (a) schematics of groove structure for meta-atom design. (b) Reflection phase and reflectance distribution as a function of the height of the grooves of the groove structure with fixed width of meta-atom $w=6mm$ and width of grooves $w_{g}=3mm$ at working frequency of 13.3 GHz.

The height of the grooves of the 4 meta-atoms for our anomalous meta-deflector with $70^{\circ}$ bending angle is listed in Table S1.

| Number of meta-atom | $h$ (mm) |
| --- | --- |
| 1 | 7.2 |
| 2 | 6.5 |
| 3 | 3.4 |
| 4 | 1.6 |

Table S1. The chosen height of the grooves for the anomalous deflector design at working frequency of 13.3 GHz.

1. **Structure design for multiple-reflected beam generator**


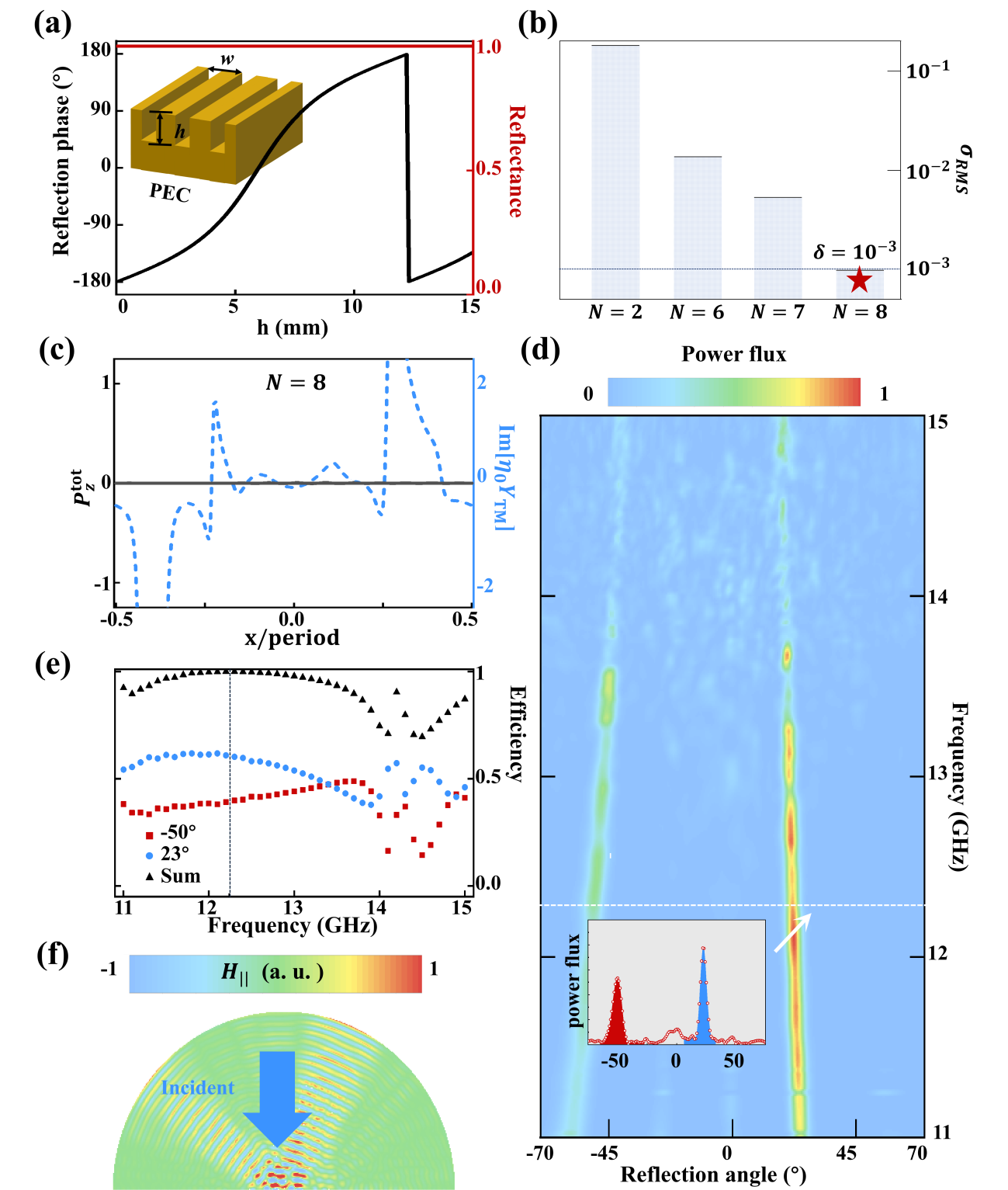


Figure S4. Design of multiple-reflected beam generator. (a) reflection phase and reflectance distribution of the meta-atom in groove structure (see inset) with fixed $w=2\mathrm{mm}$ and $w_{g}=1\mathrm{mm}$, at the working frequency of 12.24 GHz. (b) The process of minimizing the parameter $\sigma_{\mathrm{RMS}}$, here the criterion is set to $\delta={10}^{-3}$ for accuracy. (c) The real and image part of the calculated surface admittance. (d) The simulated spectrum of the designed metasurface when shined normally with Gaussian wave, while the inset shows the angular distribution at the working frequency of 12.24 GHz. (e) The calculated efficiency (black triangle), as well as the energy participant of -50° (red square) and 23° (blue circle). (f) The field distribution of the calculated system at he working frequency. Here, $P_{z}^{\text{tot}}$ is normalized with $P_{z}^{i}$.

| Number of meta-atom | $h$ (mm) | Number of meta-atom | $h$ (mm) |
| --- | --- | --- | --- |
| 1 | 2.2 | 17 | 6.2 |
| 2 | 3.4 | 18 | 5.9 |
| 3 | 3.6 | 19 | 5.7 |
| 4 | 3.3 | 20 | 5.6 |
| 5 | 2.8 | 21 | 5.6 |
| 6 | 2.5 | 22 | 5.8 |
| 7 | 2.9 | 23 | 6.3 |
| 8 | 3.3 | 24 | 8.4 |
| 9 | 3.3 | 25 | 1.2 |
| 10 | 2.1 | 26 | 1.8 |
| 11 | 7.9 | 27 | 11.6 |
| 12 | 6.1 | 28 | 7.8 |
| 13 | 5.8 | 29 | 6.7 |
| 14 | 5.8 | 30 | 6.5 |
| 15 | 6 | 31 | 7.1 |
| 16 | 6.2 | 32 | 9.8 |

Table S2. The height of the grooves of 32 meta-atoms for the meta-design of multiple-reflected beam generator shown in Figure 7 in the main-text.

**Section IV** **– Details information for Figure 5(e) in the main text**

1. **Anomalous meta-deflector cased on our approach**


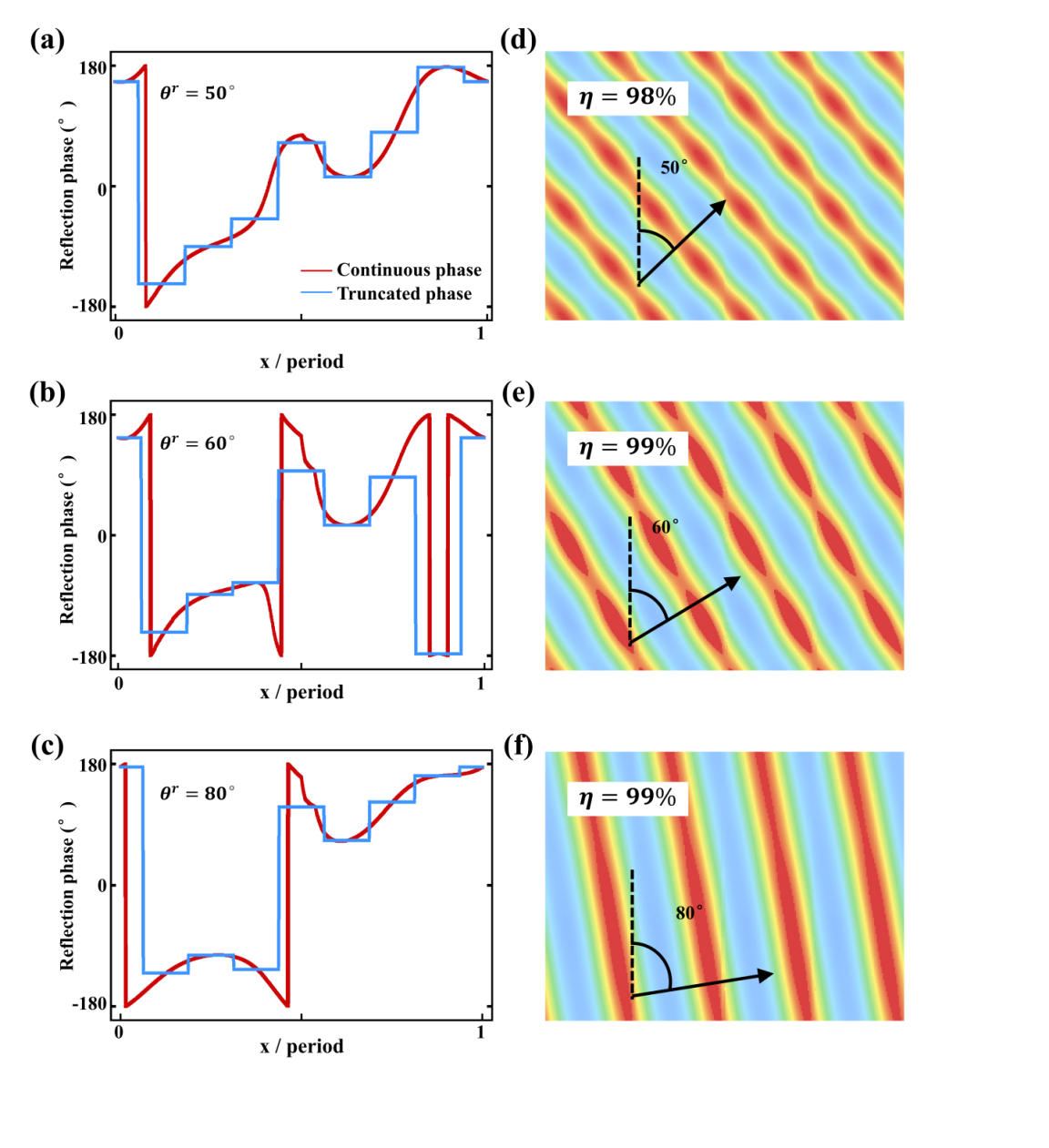


Figure S5. The calculated continuous phase profile for the perfect anomalous meta-deflectors with bending angle of (a) $50^{\circ}$, (b) $60^{\circ}$, and (c) $80^{\circ}$ and the truncated one with different groove structures. The FEM-simulated reflected H-field of designed anomalous meta-deflector with bending angle of (c) $50^{\circ}$, (d) $60^{\circ}$, and (e) $80^{\circ}$ with corresponding working efficiency of 16.3, 13.3, and 12.7 GHz.

1. **Anomalous meta-reflector based on conventional** **Huygens-principle scheme**

The reflection phase for the conventional Huygens-principle scheme is with simply linear gradient, so we only show the realistic design of anomalous reflector with bending angles of $25^{\circ}$, $35^{\circ}$, $50^{\circ}$, $60^{\circ}$, $70^{\circ}$, and $80^{\circ}$, based on only 4 meta-atoms. Here the width of the meta-atoms is set to $w=6 mm$, $w_{g}=3\mathrm{mm}$. The other parameters of the grooves and the working frequencies of the metasurfaces are shown in Table S3. Notice that according to the scaling law, all of the working frequencies can be shifted by simply changing the geometrical parameters of the meta-atoms.

|  | $25^{\circ}$ | $35^{\circ}$ | $50^{\circ}$ | $60^{\circ}$ | $70^{\circ}$ | $80^{\circ}$ |
| --- | --- | --- | --- | --- | --- | --- |
| Working frequency (GHz) | 29.57 | 21.78 | 16.31 | 14.42 | 13.29 | 12.68 |
| $h_{1}$ (mm) | 1.5 | 2.1 | 2.9 | 3.3 | 3.6 | 3.8 |
| $h_{2}$ (mm) | 2.1 | 3.0 | 4.2 | 4.8 | 5.2 | 5.5 |
| $h_{3}$ (mm) | 2.9 | 4.1 | 5.6 | 6.4 | 6.9 | 7.3 |
| $h_{4}$ (mm) | 5.1 | 6.9 | 9.2 | 10.4 | 11,3 | 11.8 |

Table S3. The parameters for the designed gradient metasurface with groove structure.

**Reference**

1. Whittaker, Edmund Taylor. "XVIII.—On the functions which are represented by the expansions of the interpolation-theory." *Proceedings of the Royal Society of Edinburgh* 35, 181-194 (1915).
2. Kotelnikov, Vladimir Aleksandrovich. "On the transmission capacity of the ‘ether’and of cables in electrical communications." *Proceedings of the first All-Union Conference on the technological reconstruction of the communications sector and the development of low-current engineering.* Moscow. 1933.
3. Shannon, Claude Elwood. "A mathematical theory of communication." *The Bell system technical journal*27(3), 379-423 (1948).
